# Implementation of stroke prevention: a review of challenges and opportunities in the Americas

**DOI:** 10.1016/j.lana.2026.101468

**Published:** 2026-05-22

**Authors:** Sheila O. Martins, Pedro Ordunez, Wyllians Vendramini Borelli, Bruce Ovbiagele, Victor C. Urrutia, Carlos Abanto, Matias J. Alet, Bassel Almarie, Tony Fabián Alvarez, Pablo Amaya, Sebastian Ameriso, Antonio Arauz, Miguel A. Barboza, Hernán Bayona, Juan Calleja, Vanessa Cano-Nigenda, Leonardo Augusto Carbonera, Rodrigo M. Carrillo-Larco, Freddy Constanzo, Angel Corredor, Ana Cláudia de Souza, Jose Danilo Diestro, Felipe Fregni, Rodrigo Guerrero, Claudio Jimenez, Fernando Lanas, Ramón Martinez, Carla Moro, Paula Muñoz-Venturelli, Victor Navia, Nelson Novarro-Escudero, Verónica V. Olavarría, Antonio Bernabé-Ortiz, Octavio Pontes-Neto, Virginia Pujol, Alejandro A. Rabinstein, Julieta Rosales, Andres Rosende, Gisele Sampaio Silva, Gustavo Saposnik, Souvik Sen, Luciano Sposato, Fernando D. Testai, Craig S. Anderson, Pablo M. Lavados

**Affiliations:** a Department of Neurology and Neurosurgery, Hospital Moinhos de Vento, Porto Alegre, Brazil; b Neurology Department, Hospital de Clínicas de Porto Alegre, Porto Alegre, Brazil; c Universidade Federal do Rio Grande do Sul, Porto Alegre, Brazil; d Department of Noncommunicable Diseases and Mental Health, Pan American Health Organization, Washington, DC, USA; e Department of Neurology, University of California San Francisco Weill Institute for Neurosciences, San Francisco, CA, USA; f Department of Neurology, Johns Hopkins University School of Medicine, Baltimore, MD, USA; g The Cerebrovascular Disease Research Center, National Institute of Neurological Sciences, Lima, Peru; h Departamento de Neurología Vascular, Centro Integral de Neurología Vascular, Fleni, Ciudad Autónoma de Buenos Aires, Argentina; i Hospital General de Agudos J. M. Ramos Mejía, Ciudad Autónoma Buenos Aires, Argentina; j Neuromodulation Center and Center for Clinical Research Learning, Spaulding Rehabilitation Hospital and Massachusetts General Hospital, Harvard Medical School, MA, USA; k Centro de Excelencia en ACV, Instituto Neurológico, Hospital Internacional de Colombia-FCV, Colombia; l Stroke Program, Neurology Department, Fundación Valle del Lili, Cali, Colombia; m Instituto Nacional de Neurología y Neurocirugía Manuel Velasco Suárez, México City, Mexico; n Departamento de Neurociencias, Hospital Dr. Rafael A. Calderón Guardia, Universidad de Costa Rica, San José, Costa Rica; o Universidad de los Andes School of Medicine, Bogotá, Colombia; p Stroke Center, Hospital Simón Bolívar, Subred Norte, Bogotá, Colombia; q Emory Global Diabetes Research Center and Hubert Department of Global Health, Rollins School of Public Health, Emory University, Atlanta, USA; r Neurology Unit, Hospital Las Higueras, Talcahuano, Chile; s School of Medicine, Universidad Católica de la Santísima Concepción, Concepción, Chile; t Department of Neurology, Stroke Center, Clínica Central del Quindío, Armenia, Colombia; u Unity Health - St. Michael's Hospital, Li Ka Shing Knowledge Institute, University of Toronto, Toronto, Canada; v Division of Neurology, Department of Medicine, University of Toronto, Toronto, Ontario, Canada; w Harvard T.H. Chan School of Public Health, Harvard University, MA, USA; x Department of Neurology, Neurosurgery and Interventional Neuroradiology, Clínica Santa María, Santiago, Chile; y Department of Internal Medicine, Faculty of Medicine, Universidad de la Frontera, Temuco, Chile; z Departamento de Neurologia, Hospital Municipal São José, Joinville, SC, Brazil; aa Centro de Estudios Clínicos, ICIM, Facultad de Medicina Clínica Alemana Universidad del Desarrollo, Santiago, Chile; ab Unidad de Neurología, Hospital Padre Hurtado, Santiago, Chile; ac Primary Stroke Center, Pacífica Salud, Hospital Punta Pacífica, Panamá, Panama; ad Servicio de Neurología, Departamento de Neurología y Psiquiatría, Clínica Alemana de Santiago, Facultad de Medicina Clínica Alemana Universidad del Desarrollo, Santiago, Chile; ae Faculty of Health Sciences, Universidad Científica del Sur, Lima, Peru; af Department of Neuroscience and Behavioral Sciences, Ribeirão Preto Medical School, University of São Paulo, Ribeirão Preto, Brazil; ag Department of Neurology, Mayo Clinic, Rochester, MN, USA; ah Servicio de Neurología Vascular, Departamento de Neurología, Clínica La Sagrada Familia, Buenos Aires, Argentina; ai Neurology Department, Universidade Federal de São Paulo (UNIFESP) and Albert Einstein Hospital, São Paulo, Brazil; aj Stroke Outcomes & Decision Neuroscience Research Unit, Department of Medicine, University of Toronto, Toronto, Canada; ak Department of Neurology, University of South Carolina School of Medicine, Prisma Health Medical Group Midlands, Columbia, SC, USA; al CNS Dept. K. & Dr. Henry Barnett Chair in Stroke Research, Schulich School of Medicine & Dentistry, Western U. Director. Heart & Brain Lab, London, ON, Canada; am Department of Neurology and Rehabilitation, University of Illinois Chicago College of Medicine, Chicago, IL, USA; an The George Institute for Global Health, Faculty of Medicine, University of New South Wales, Sydney, Australia; ao Institute for Science and Technology for Brain-Inspired Intelligence, Fudan University, China

**Keywords:** Primary prevention, Secondary prevention, Stroke, Implementation, Cardiovascular disease

## Abstract

Stroke remains a leading cause of death and disability throughout the Americas, disproportionately impacting low-and middle-income countries and underserved populations. In this review, we examine the status of stroke prevention in the Americas. Prevention is essential, yet unequal access to healthcare has led to major disparities - especially among rural populations, ethnic minorities, and lower socioeconomic status. Models like the WHO HEARTS Program demonstrate that evidence-based programs can be tailored to local contexts. Telehealth and digital tools play a critical role in empowering patients, educating communities, and supporting healthcare workers. Despite growing efforts, challenges persist due to health inequities, gaps between policy and implementation, and underinvestment. Strengthening prevention will support countries in achieving the United Nations Sustainable Development Goals, targeting a one-third reduction in premature deaths from non-communicable diseases by 2030. This paper outlines effective strategies for implementing stroke prevention, emphasizing healthy lifestyles, early detection of risk factors, and system-level interventions.

## Resumo

O Acidente Vascular Cerebral (AVC) permanece como uma das principais causas de morte e incapacidade em todas as Américas, impactando desproporcionalmente os países de baixa e média renda e as populações vulneráveis. Nesta revisão, examinamos o estado atual da prevenção do AVC nas Américas. A prevenção é essencial; contudo, o acesso desigual aos cuidados de saúde resultou em disparidades significativas – especialmente entre populações rurais, minorias étnicas e indivíduos de baixo nível socioeconômico. Modelos como o Programa HEARTS da OMS demonstram que programas baseados em evidências podem ser adaptados aos contextos locais. A telessaúde e as ferramentas digitais desempenham um papel fundamental na capacitação dos pacientes, na educação das comunidades e no apoio aos profissionais de saúde. Apesar dos esforços crescentes, os desafios persistem devido às iniquidades em saúde, às lacunas entre a política e a implementação, e ao subfinanciamento. O fortalecimento da prevenção apoiará os países no alcance dos Objetivos de Desenvolvimento Sustentável das Nações Unidas, visando uma redução de um terço nas mortes prematuras por doenças crônicas não transmissíveis até 2030. Este artigo apresenta estratégias eficazes para a implementação da prevenção do AVC, enfatizando estilos de vida saudáveis, a detecção precoce de fatores de risco e intervenções em nível sistêmico.

## Abstract (es)

El ataque cerebrovascular sigue siendo una de las principales causas de muerte y discapacidad en las Américas, afectando de manera desproporcionada a los países de ingresos bajos y medianos, así como a las poblaciones desatendidas. En esta revisión, examinamos el estado de la prevención del accidente cerebrovascular en las Américas. La prevención es esencial; sin embargo, el acceso desigual a la atención sanitaria ha provocado disparidades importantes, especialmente entre las poblaciones rurales, las minorías étnicas y los grupos de nivel socioeconómico bajo. Modelos como la Iniciativa HEARTS de la OMS demuestran que los programas basados en evidencia pueden adaptarse a los contextos locales. La telesalud y las herramientas digitales desempeñan un papel fundamental en el empoderamiento de los pacientes, la educación de las comunidades y el apoyo a los trabajadores de la salud. A pesar de los crecientes esfuerzos, persisten los desafíos debido a las inequidades en salud, las brechas entre la política y la implementación, y la inversión insuficiente. El fortalecimiento de la prevención apoyará a los países en el logro de los Objetivos de Desarrollo Sostenible de las Naciones Unidas, con la meta de reducir en un tercio las muertes prematuras por enfermedades no transmisibles para el año 2030. Este artículo describe estrategias efectivas para implementar la prevención del accidente cerebrovascular, enfatizando los estilos de vida saludables, la detección temprana de factores de riesgo y las intervenciones a nivel sistémico.

## Introduction

Stroke is a major health issue in the Americas, significantly contributing to morbidity and mortality. In 2023, there were 1.1 million new cases and 12.9 million people living with stroke. During this period, 500,000 deaths occurred, resulting in 11.4 million disability-adjusted life years (DALYs) due to stroke.^1^^,^^2^ The region faces unique challenges stemming from its diverse populations, socioeconomic inequalities, and differences in healthcare access and quality, both among and within countries. Additionally, the demographic transition in recent decades has shifted from a high impact of infectious diseases to an increased incidence and mortality from cardiovascular diseases (CVDs), including strokes. Consequently, stroke was the second-leading cause of death regionwide from 1990 to 2019, following ischemic heart disease. However, COVID-19 emerged as the primary cause of death during 2020–2021.

As a leading cause of death and disability in the Americas, there is a need to examine the current epidemiology of stroke and its risk factors, and outline effective strategies for implementing stroke prevention and treatment in the region, emphasizing the promotion of healthy lifestyles, early detection of risk factors, and system-level interventions. This is the second of two narrative review articles examining the status of stroke prevention in the Americas. In this article, we first briefly highlight the epidemiology of stroke, including demographic changes and stroke burden outcomes, its main risk factors, and healthcare disparities across countries of the region. Secondly, we outline the needs and gaps in stroke prevention, emphasizing the need to address social determinants of health, promoting healthy lifestyles and reducing exposure to modifiable risk factors, strengthening healthcare systems, education and awareness. Then, we underscore the implementation of effective strategies for stroke primary prevention in the Americas, and outline challenges and future directions for stroke prevention, treatment and control.

## Search strategy and selection criteria

This narrative review explored the implementation, evaluation and impact of primary prevention strategies for stroke in the Americas. The search strategy focused on identifying relevant literature from OVID Medline and LILACS (Latin American and Caribbean Literature on Health Sciences), for primary stroke and cardiovascular disease prevention research published between January 2010 and May 2025 because of the recent advances in preventive strategies in public healthcare services. The following keywords were searched in the title or abstract: (“stroke”, “cerebrovascular disease”, “ischemic heart disease” or cardiovascular disease”, “risk factor”, “hypertension”, “diabetes”) AND (“prevention”, “guidelines”, “incidence”, “prevalence”, “implementation”, “burden”) AND (“Latin America”, “America”, “United States”, “Canada”, “South America”, “Central America”, “North America”). Key terms were combined according to the Boolean operators in each database searched. No language restrictions were applied. Additionally, we manually searched the reference lists of relevant publications and consulted with experts in stroke, cardiovascular disease, and other relevant stakeholders to complement the electronic searches, including the monitoring documents from the World Health Organization and the Pan American Health Organization.

## Understanding the burden of stroke in the Americas

### Demographic and epidemiologic changes

Recent decades have seen major developments in the Americas. The US and Canada remain high-income countries, while Latin American and Caribbean nations improved access to education, water, sanitation, healthcare, affordable technologies, and immunizations, helping control many communicable diseases.^3^ Urbanization has led to nearly 90% of people living in cities, increasing risk factors for cardiovascular, renal, and metabolic diseases, along with rises in stroke and heart attack morbidity and mortality.^4^

**Fig. 1:**
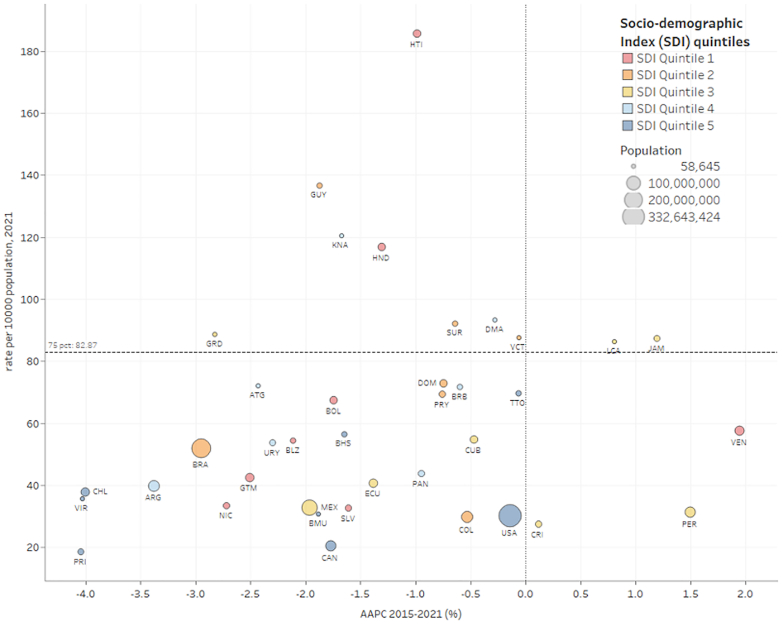
**Age-standardized stroke mortality in 2021 against their average annual percent change from 2015 to 2021 across countries of the Americas.** Notes: Data are shown the age-standardized stroke mortality rates per 100,000 population in 2021 against their average annual percent change (AAPC) in the period 2015–2021 across countries of the Americas. Each dot represents a country, whose size is proportional to the population size, and the color identifies the socio-demographic index (SDI) quintile. Source: Global Burden of Disease Study 2021 and Martinez R et al.^3^

Age-standardized incidence, prevalence, mortality, DALYs, Years Lived with Disability (YLDs), and Years of Life Lost (YLL) rates for stroke varied widely across the Americas from 1990 to 2021. In 2021, stroke mortality averaged 38.1 per 100,000, ranging from 185.6 in Haiti to 18.5 in Puerto Rico (Fig. 1). Although most countries showed declines from 1990 to 2021, trends stagnated or reversed after 2015, with mortality increasing in five countries (Fig. 1). From 2015 to 2021, incidence rose across most age groups, with a notable increase in ischemic stroke mortality and DALYs among younger populations.^4^

### Risk factors

**Fig. 2:**
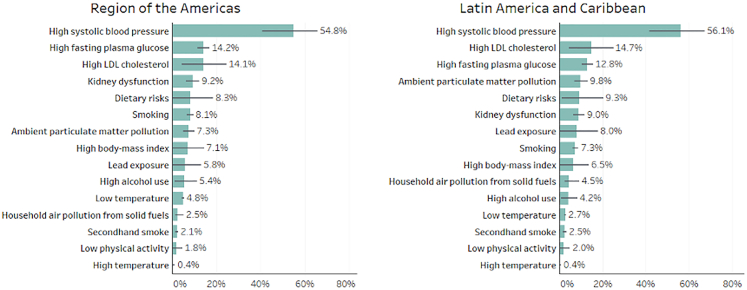
**Age-standardized death rates due to stroke attributable to leading risk factors in the Region of the Americas, and Latin America in 2021.** Notes: Data are shown as percentage of population attributable fraction of the stroke deaths due to the top 15 risk factors. Black horizontal line represents the 95% uncertainty interval of the estimate. Source: Global Burden of Disease Study 2021.^1^

Metabolic factors like hypertension, high LDL cholesterol, elevated fasting glucose, and obesity are major contributors to stroke in the Americas,^5^ with hypertension responsible for 54.8%.^5^, ^6^, ^7^ While risks are similar to high-income countries, poorer control increases the burden.^8^ High LDL-cholesterol affects 20% of the Latin American population and causes ∼7900 stroke deaths in the Americas in 2023 (Fig. 2).^2^^,^^9^ Behavioral risks—tobacco, unhealthy diet, physical inactivity—significantly influence stroke, accounting for over 8.1%, 8.3%, and 2% of deaths, respectively, with alcohol playing a smaller role (Fig. 2).^1^^,^^6^^,^^10^^,^^11^ Oral infections, especially periodontal disease, promote inflammation and may raise stroke risk, especially in disadvantaged groups. Recent evidence supports oral health as a modifiable, low-cost target for primary stroke prevention.^12^ Environmental factors like air pollution (2.5 μm, or PM_2.5_) cause about 7.3% of stroke deaths in the Americas^13^; extreme temperatures and toxins also increase danger, especially in lower-income countries.^1^

### Healthcare disparities

Access to healthcare differs significantly between and within countries, resulting in disparities in stroke prevention, diagnosis, treatment, and outcomes, especially among racial and ethnic minorities, lower socioeconomic groups, and rural populations. Lower income, education, and health literacy are associated with a higher prevalence and poorer management of risk factors like hypertension, diabetes, obesity, smoking, and physical inactivity, all of which are worsened by limited preventive care access.^14^ Rural populations face additional challenges, including fewer providers and longer travel distances, contributing to higher morbidity and mortality.

In the Americas, stroke disproportionately affects marginalized groups. In the United States, women and Black and Hispanic communities face inequities in prevention and risk management, leading to higher incidence and worse outcomes.^15^^,^^16^ In Latin America and the Caribbean, the stroke burden is influenced by socioeconomic inequalities, urban growth, and widespread modifiable risk factors, while limited resources restrict the effective implementation of prevention.^10^^,^^17^ Structural inequities, including systemic racism and unequal distribution of wealth and education, further increase risk.^18^^,^^19^ Globally, lower-income regions face higher stroke incidence and mortality due to greater risk factor burden and limited access to preventive care.^1^^,^^20^

## Needs and gaps in primary prevention

### Addressing social determinants of health

Addressing disparities in stroke care requires comprehensive strategies that involve improving access to high-quality primary healthcare services, enhancing patient education, and implementing community-based interventions tailored to the specific needs of disadvantaged populations, along with advocating for policies that promote health equity.^16^^,^^17^^,^^21^, ^22^, ^23^ To reduce disparities in access to primary prevention, several strategies can be implemented.

#### Team-based care, community health-workers and community-engaged interventions

Team-based care transforms stroke prevention from a fragmented, individual effort to a coordinated, comprehensive, and patient-centered approach, leading to better health outcomes and a more equitable distribution of care. Instead of a doctor or specialist performing every aspect of a patient's care, specific tasks that can be safely and effectively performed by other well-trained healthcare workers (but still adequately trained) are shifted to them, in accordance with their scope of practice. Shifting tasks is a crucial strategy employed to address workforce shortages, enhance access to care, and boost efficiency, particularly in resource-constrained settings.^24^^,^^25^

Community engagement is crucial. Engaging Community Health-Workers can bridge gaps in care by offering culturally and linguistically appropriate support, education, and navigation through the healthcare system, thereby improving access to preventive care, particularly in underserved areas.^26^ The American Heart Association and American Stroke Association emphasize that community health-workers, as part of multidisciplinary teams, improve patient follow-up, medication adherence, risk-factor education, and the management of social determinants of health related to stroke risk.^24^ Across the Americas, this team-based approach has been implemented in low-, middle-, and high-income countries to improve population awareness of vascular risk factors and to strengthen blood pressure control, diabetes management, and tobacco cessation—core pillars of effective stroke prevention. It has been shown to improve self-management and medication adherence, especially among Black, Hispanic, and Latino populations.^24^

The Heart Outcomes Prevention and Evaluation 4 (HOPE 4), conducted in Colombia and Malaysia, was a cluster-randomized controlled trial that aimed to evaluate a comprehensive, community-based intervention for individuals with newly diagnosed or poorly controlled hypertension.^27^^,^^28^ The intervention involved non-physician health-workers using tablet-based management algorithms, providing free antihypertensive and statin medications (recommended by non-physician health-workers and supervised by physicians), and including a family member or friend as a treatment supporter to promote adherence and healthy behaviors. After 12 months, the intervention group demonstrated a greater reduction in LDL-cholesterol, blood pressure control (<140/90 mmHg) achieved in 69% versus 30% in the control group, and a 4.8% reduction in estimated 10-year CVD risk compared to usual care (95% CI, −7.11 to −2.44; p < 0.0001). As no safety concerns were noted, these findings suggest that a comprehensive, team-based approach, led by non-physician health-workers and supported by family, can significantly improve hypertension, hyperlipidemia, and reduce cardiovascular risk in resource-limited settings.

#### Culturally tailored interventions

Developing and increasing access to culturally tailored health education, nutritional counseling, and preventive services —based on evidence but adapted to local realities—can address the specific needs of minority populations. Media campaigns and health messages in the preferred languages of target populations can also enhance engagement and effectiveness.^16^^,^^29^

#### Workforce diversity and training

Increasing the diversity of the healthcare workforce can improve the quality of care and reduce disparities. Diverse healthcare teams are better equipped to understand and address the unique needs of underrepresented populations.^30^ Training all teams (doctors, nurses, other health professionals, and community health-workers) in the protocols and pathways for primary and secondary prevention is essential to ensure the highest quality of care and the best outcomes.

#### Telehealth and mobile health units

Expanding telehealth services and deploying mobile health units can improve access to preventive care in underserved and rural areas. These technologies facilitate access to specialists who support primary care clinicians in diagnosis and treatment, allowing for chronic disease management with direct contact with patients and reducing barriers related to distance and transportation.^24^^,^^31^

The implementation of teleneurology programs could address issues related to the lack of access to specialists in different regions. These programs adequately respond to most outpatient conditions that increase the burden of neurological diseases, particularly stroke. Additionally, they can easily identify which patients require continued follow-up via telemedicine and which should be referred to tertiary healthcare facilities.

In Latin America, a teleneurology program has been described that operates in a synchronous mode for outpatient consultations. This program has successfully enabled remote care for outpatients at home, as well as at local primary and secondary facilities.^32^ It has been shown to improve access to neurological care by neurologists, demonstrating a high level of user satisfaction while reducing the waiting time for the first consultation.^33^ The program also facilitates timely follow-up appointments for various neurological conditions, including stroke patients after hospitalization, all while maintaining local legal compliance.^34^

#### Community-engaged interventions and digital technologies

The FAITH! App Pilot Study (Fostering African American Improvement in Total Health) is an important example. This study collaborated with Black churches to co-create a mobile health intervention targeting multiple CVD risk factors, highlighting the effectiveness of community engagement in enhancing health behaviors and promoting sustainability.^24^ The Stroke Riskometer is a powerful, free smartphone App translated into 30 languages, designed to assess an individual's risk of stroke within the next 5–10 years.^35^^,^^36^ Developed by researchers at AUT University in New Zealand, it is based on a questionnaire that focuses on identifying and recommending modifications to lifestyle risk factors, making it a valuable resource for both individuals and healthcare professionals in promoting stroke prevention.

#### Policy interventions

The inclusion of cardiovascular prevention in each health policy is crucial to ensure equitable access to the most effective, evidence-based treatments. The core strategies include population-level policy interventions (such as tobacco taxation and control, elimination of trans fats, and compulsory salt reduction in manufactured foods), primary prevention and health promotion, systematic screening, evidence-based treatment protocols, access to essential medicines, team-based care, and robust monitoring systems. The medications should be either low-cost or, ideally, covered by insurance or public health programs, making it easier for patients to obtain and adhere to necessary treatments.

Enhancing Affordable Care Act insurance subsidies and expanding Medicaid access have effectively reduced disparities in preventive service utilization and improved cardiovascular outcomes in the United States. Policies should also emphasize the collection and analysis of data by race and ethnicity to identify and address inequities.^37^

### Promoting healthy lifestyles and addressing modifiable risk factors

Adherence to multiple healthy lifestyle behaviors reduces cardiovascular risk by 66%, stroke risk by 60%,^38^ and mortality by 67%,^39^ highlighting the importance of comprehensive prevention strategies.^40^ Although preventable stroke risk factors are known, implementing prevention strategies remains challenging.

Regular moderate-to-vigorous physical activity (≥150 min/week) can reduce stroke risk by up to 25%.^41^ In Latin America, urban designs that favor cars, socioeconomic constraints, and safety issues hinder adherence to physical-activity. Community exercise programs, such as walking clubs and low-cost aerobics, enhance physical activity.^42^^,^^43^ Scaling these interventions requires collaboration to improve public spaces, subsidize classes, and incorporate physical-activity counseling into primary care.

Healthy dietary patterns, including DASH and Mediterranean diets, are associated with lower stroke risk,^44^, ^45^, ^46^ although food insecurity and cost remain major barriers. Policies such as food subsidies, front-of-pack labeling, and taxes on sugary drinks have shown promise in promoting healthier choices as demonstrated in Peru, Mexico, and Chile.^47^^,^^48^ Smoking cessation can rapidly reduce this risk within a few years,^49^ supported by counseling and pharmacotherapy (nicotine replacement therapy, varenicline or bupropion).^49^^,^^50^ Reducing harmful alcohol use through screening, brief interventions, and referrals can lower stroke risk.^51^ Public health policies—such as tobacco taxation, advertising bans, and alcohol restrictions reduce risk factors at the population level.

A trial in Argentina showed that community health–worker interventions significantly reduced projected 10-year stroke risk.^52^ To maximize impact in the Americas, programs should be culturally tailored, address socioeconomic barriers, and be sustainably funded through public–private partnerships or supportive policies promoting access to healthy foods and safe physical activity, with ongoing evaluations.

**Fig. 3:**
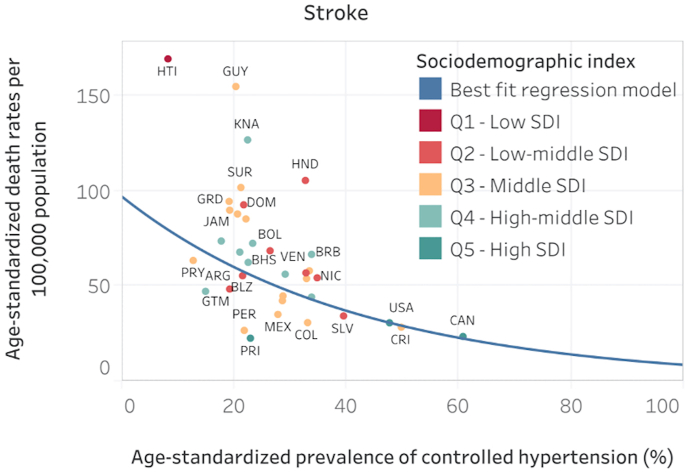
**Death rates caused by stroke in American countries according to the prevalence of controlled hypertension.** Abbreviations: ARG - Argentina; BHS - Bahamas; BLZ - Belize; BOL - Bolivia; BRA - Brazil; BRB - Barbados; CAN - Canada; CHL - Chile; COL - Colombia; CRI - Costa Rica; CUB - Cuba; DOM - Dominican Republic; ECU - Ecuador; SLV - El Salvador; GRD - Grenada; GTM - Guatemala; GUY - Guyana; HTI - Haiti; HND - Honduras; JAM - Jamaica; KNA -Saint Kitts and Nevis; LCA - Saint Lucia; MEX - Mexico; NIC - Nicaragua; PAN - Panama; PER - Peru; PRI - Puerto Rico; PRY - Paraguay; SUR - Suriname; TTO - Trinidad and Tobago; URY - Uruguay; USA–United States of America; VEN - Venezuela. Source,^55^ reproduced with permission.

Effective control of metabolic risk factors is essential. Hypertension remains the leading modifiable risk factor, with a 10 mmHg reduction in systolic blood pressure lowering stroke risk by about 27%.^48^^,^^53^ However, control rates remain suboptimal in many countries due to medication costs, inadequate follow-up, therapeutic inertia, and low adherence.^54^ Strategies such as single-pill combinations, community health-worker programs, and improved access to affordable medications can enhance control. Fig. 3 shows that American countries with better control of hypertension have lower rates of deaths related to stroke.

Availability of statins, insulin, and essential oral diabetes therapies should be ensured, and long-acting antihypertensives in public health settings can improve adherence through once-daily dosing. Access to cardioprotective glucose-lowering agents—such as SGLT2-inhibitors and GLP-1 receptor agonists—can further reduce mortality and cardiovascular events, although disparities in access persist.^18^^,^^56^^,^^57^ Expanding universal health coverage, implementing task-shifting models for chronic disease management, implementing continuous education to health professionals, and leveraging telemedicine for remote monitoring can help bridge these gaps. Ultimately, a comprehensive, multidisciplinary approach to metabolic risk factor treatment is vital for reducing the burden of stroke across the Americas.

Addressing environmental risk factors, such as air pollution from very fine particulate matter and solid fuels, substance poisoning, among others,^54^^,^^58^ should primarily involve policymakers and governmental actions. This necessitates a comprehensive approach that encompasses assessment, planning, implementation, and monitoring. Sustainable practices should be promoted by stakeholders responsible for setting limits on emissions, regulating waste disposal, and protecting natural habitats.

### Strengthening healthcare systems

Healthcare systems in the Americas face challenges in prevention and chronic disease management. Strengthening health policies to reduce access disparities is key to lowering stroke incidence. A key strategy is developing a National Plan for Stroke,^59^ integrated into the Universal Health Coverage package whenever a universal healthcare system is available. This plan includes prevention, acute care, and rehabilitation, along with specific steps and a timeline for implementation, emphasizing an integrated stroke care pathway to promote value-based stroke care.^60^ To be feasible, the plan should enhance community-based interventions and services and financial protection that ensure equitable access to healthcare. This includes expanding the availability of primary care services, such as extended hours to late night and weekends, team-based care, and telemedicine to reach remote areas. Continuous education and capacity building, always evidence-based and following the guidelines,^18^^,^^61^ paired with streamlined treatment protocols and a comprehensive clinical pathway, can enhance the skills of healthcare professionals^27^ and ultimately improve implementation effectiveness.^60^^,^^62^

The policies to promote health equity should include public health campaigns and partnerships, enhanced food labeling, reduced sodium intake, and encouraged physical activity in schools and workplaces.^26^ Campaigns in schools to educate children about healthy lifestyles and stroke signs should begin early. Monitoring systems that assess the prevalence and control of risk factors are fundamental. Quality monitoring can be a strategic approach for implementing primary stroke prevention in America by leveraging data-driven methodologies to improve clinical practice and patient outcomes.

The Latin American Stroke Ministerial Meetings have shown that collaborative efforts between medical societies and governments can significantly improve stroke care infrastructure and the implementation of national plans for stroke.^63^^,^^64^

### Stroke center development and certification

The Stroke Center, defined as a hospital in which there is an organization of stroke care from screening, diagnosis, treatment to recovery; via policies and protocols of care and data collection for quality improvement, is a resource that can have a significant impact not only in acute treatment and recovery of stroke but also in primary and secondary prevention.^65^

Stroke Centers have been recognized as a driver of good outcomes in stroke care^66^ and are part of the WHO Global Action Plan for Non-communicable Diseases. Since the introduction of certification programs in the United States in 2003, Stroke Centers have proliferated, improving stroke outcomes.^67^^,^^68^ This has followed in other regions in the world, including in Latin America, led by the Ibero-American Society of Cerebrovascular Diseases (SIECV) and World Stroke Organization in 2021.^67^^,^^68^

The Stroke Center is the building block of the stroke system of care and plays a definitive role in organizing care not only within the hospital, but also in the community. It provides education on primary and primordial prevention, acute treatments, and rehabilitation. It delivers primary and secondary prevention, leads stroke care locally and regionally, and helps shape public policy for stroke prevention.

### Stroke awareness campaigns

In recent years, awareness campaigns about stroke signs, risk factors, and prevention have been launched across the Americas.^26^ Between 2018 and 2020, public awareness of stroke increased from 25% to 75% among 13 countries in the region. These campaigns can empower individuals to take proactive steps in managing their health and seeking timely medical intervention.^63^

There are two examples of stroke awareness campaigns regarding risk factors control in the Americas. Firstly, the American Heart Association’s 2024 guidelines emphasize the “Life’s Essential 8” - a set of actionable behaviors and health factors including healthy diet, physical activity, tobacco cessation, healthy sleep, weight management, and control of blood pressure, cholesterol, and blood sugar—as key to preventing up to 80% of strokes.^18^ Healthcare providers who understand and use LE8 daily may ultimately improve identification and treatment of 8 stroke risk factors. Secondly, the World Stroke Organization has a strong awareness campaign with significant participation from Latin American countries^69^ with important impact regionally, such as the “World Stroke Day” involving different levels of healthcare providers. These campaigns help raise awareness and educate both patients and healthcare personnel.

## Implementing primary prevention in the Americas

**Fig. 4:**
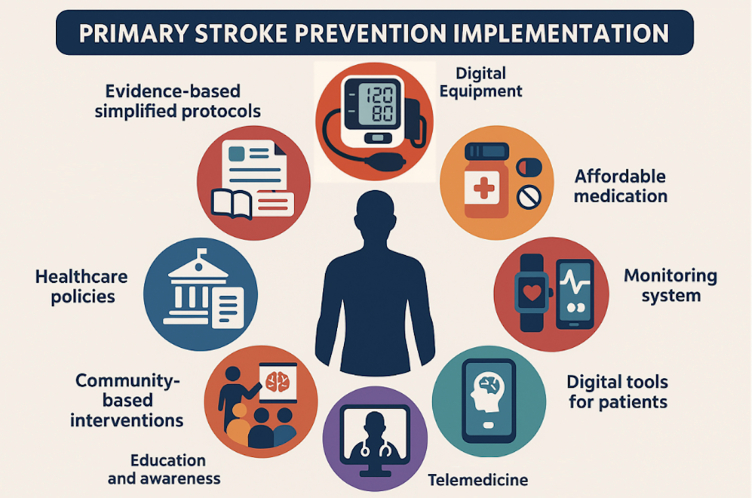
**Multicomponent approach to primary stroke prevention implementation**.

The implementation of effective strategies for stroke prevention in the Americas requires a multifaceted approach, considering regional particularities and socioeconomic challenges. Many levels of responsibility are necessary to engage together towards a single aim. These levels include policymakers, community organizations, and individual measures. The collaborative efforts of governments, experts, and scientific societies in Latin America have enabled the creation of national stroke action plans, encompassing prevention, treatment, and rehabilitation, with the goal of mitigating its impact in the region.^63^ Table 1 presents the steps for implementing stroke prevention in public health, and Fig. 4 shows the main elements to be implemented.^25^^,^^58^^,^^63^^,^^64^^,^^70^

**Table 1:** Steps for implementing primary stroke prevention.

Action	Description	Responsible
Situational evaluation	Evaluate: - Number and distribution of PCU∗∗ - Structure of PCU∗∗: infrastructure, equipment, personnel (who is on the team? Number of doctors, nurses, nurse technicians, dentists, physiotherapists, CHWs∗, social workers, pharmacists, and others) - Medication available, ideally at no cost for patients - Patient pathways	Local health manager
Development of the treatment protocols	Implement simplified, evidence-based protocols for triaging risk factors, providing treatment with available medications, advising on lifestyle changes, and establishing goals to achieve. SPC^#^ can help improve adherence to treatments.	Specialists along with health managers Health managers, doctors
Establishing the clinical pathway	Implement a team-based care model that utilizes a patient-centered approach and task shifting among well-trained professionals guided by a standardized clinical pathway. Establish a systematic screening for hypertension and diabetes. This can start implementing a routine to measure the blood pressure of all patients assisted in the PCU∗∗ whenever possible. Include risk stratification at least once a year to adjust medication and goals. Schedule the follow-up visits based on the patients' cardiovascular risk and the goals achieved.	PCU∗∗ Leadership
Equipment	Clinically validated automatic blood pressure devices, scales, glucometers, accessible cholesterol tests, glycated hemoglobin tests, and INR measurements, among others.	PCU∗∗ Leadership
Monitoring system	Establishing systems to monitor patient outcomes and evaluate program performance facilitates continuous quality improvement. Structured electronic medical records (patient files) can facilitate information entry, collect data in a databank, and generate quality monitoring dashboards.	National or local health manager and IT people
Digital tools for patients	They can help empower patients to recognize stroke risks and risk factors while educating them on how to modify these risks (for example, the Stroke Riskometer App, translated into 30 languages). Community Health Workers (CHWs) can use it to teach the population about strokes. Following up with patients (after PCU appointments or after hospital discharge) through a smartphone app can connect health professionals with patients, evaluate patient status, access treatments and rehabilitation services, and anticipate PCU visits. This strategy can facilitate patient navigation through different levels of the healthcare system, increasing access to services.	Health managers, PCU∗∗ teams, CHWs∗ Stroke Unit Leadership, health managers, stroke societies
Telemedicine	Primary care clinicians should have access to specialists when needed, especially for more complex cases; nurses should discuss cases in areas where doctors are unavailable. For patient consultations with nurses or doctors, or for exam follow-ups (for example, anticoagulated patients to review INR)	Health managers, PCU∗∗ leadership, doctors in the PCU, Stroke Societies
Community-based interventions	CHWs can serve as a link between the PCU and the population, assisting patients in navigating the healthcare system. They can collaborate with the community to raise awareness about lifestyle modifications, treatment adherence, recognizing stroke signs, and the importance of seeking care at a stroke center.	CHW∗, PCU∗∗, stroke centers
Education	It is fundamental to educate all levels of care using evidence-based treatment protocols and clinical pathways It is important to educate the population about risk factors and stroke signs, including children in schools	Medical societies, stroke centers, and the private sector collaborating with government and local health managers
Integrated healthcare services and stakeholders	Collaboration among medical societies, governments, and the private sector is crucial for implementing primary and secondary prevention strategies, including education, surveillance, and registries for non-communicable diseases. Integrating various levels of care (primary care, stroke centers, rehabilitation services) is essential to ensure that patients transition through different complexities of care based on their needs.	Medical societies, stroke centers, and the private sector collaborating with government and local health managers Local health managers
National Plan for Stroke	A national policy for stroke, with the strategy included in the Universal Health Coverage packages, can facilitate large-scale coordinated implementation across the country. The plan should cover prevention, acute care, and rehabilitation. Financial protection and incentives are critical components of the plan. If it is not possible to implement at the national level, the plan can alternatively be implemented at the state or city level.	Government, Ministry of Health, specialists

CHW∗ = community health workers; PCU∗∗ = primary care units; SPC^#^ = single pill combination.

### WHO monitoring of noncommunicable diseases in the Americas

The WHO Monitoring of noncommunicable diseases (NCDs) in the Americas^71^ involves tracking and analyzing data related to NCDs such as CVDs, cancer, chronic respiratory diseases, and diabetes. This monitoring is crucial for understanding trends, prevalence, risk factors, and impact of these diseases in the region. It helps in formulating public health policies, allocating resources, and implementing strategies to prevent and control NCDs. The monitoring provides data on 22 indicators for the 35 Member States of the Pan American Health Organization (PAHO) regarding the recommended interventions, referred to as the World Health Organization (WHO) “Best Buys”.

The monitoring process typically includes: (1)Gathering data on the incidence, prevalence, and mortality rates of NCDs; (2)Tracking risk factors such as tobacco use, unhealthy diet, physical inactivity, and harmful use of alcohol; (3)Evaluating the capacity of health systems to respond to NCDs, including access to essential medicines and technologies; (4)Assessing the implementation of policies and interventions aimed at reducing the burden of NCDs and (5)Regularly reporting on progress towards achieving global and regional targets. The WHO collaborates with countries in the Americas to strengthen their health systems, improve data quality, and enhance the capacity to monitor and respond to NCDs effectively.

**Fig. 5:**
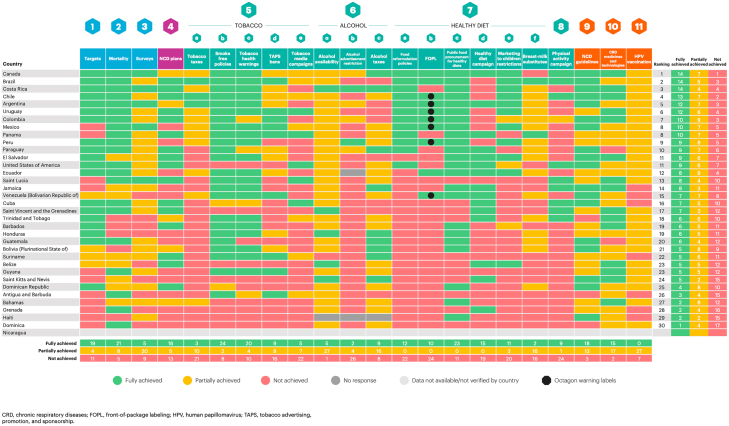
**Non-communicable diseases implementation progress in the Americas according to the WHO recommendations.** Reproduced from Progress Monitor 2025.^71^

The monitoring system ranks countries based on the total number of fully achieved indicators, highlighting the top-ranking countries: Canada, Brazil, Costa Rica, Chile, Argentina, and Uruguay, all of which have achieved 12 (55%) or more progress indicators. Most are high-income countries, except for Brazil and Costa Rica, which are middle-income countries, indicating that implementing best practices is feasible. Fig. 5 illustrates the progress of quality indicators in 2025, as recommended by the WHO to combat NCDs and implemented in American countries.

### The WHO HEARTS program: concepts, strategies, tools

The World Health Organization (WHO) HEARTS program is a global initiative aimed at reducing the burden of CVDs by strengthening primary healthcare systems. It is part of the broader Global Hearts Initiative, launched in collaboration with the United States Centers for Disease Control and Prevention (CDC) to support governments in preventing and controlling CVDs, with a particular focus on hypertension management.^70^

The HEARTS program is structured around a technical package that includes several key components designed to be implemented at the primary healthcare level. These components are:

1. Healthy Lifestyle Counseling: Encouraging lifestyle modifications such as diet, physical activity, and smoking cessation to reduce CVD risk factors.
2. Evidence-Based Treatment Protocols: Implementing standardized treatment protocols to ensure consistent and effective management of hypertension and other CVD risk factors.
3. Access to Essential Medicines and Technology: Ensuring the availability of essential medications and diagnostic tools necessary for effective CVD management.
4. CVD Risk-Based Management: Utilizing risk stratification tools to tailor interventions based on individual patient risk profiles.
5. Team-Based Care: Promoting a collaborative approach to care delivery, involving various healthcare professionals to optimize patient outcomes.
6. Systems for Monitoring and Evaluation: Establishing robust systems for tracking patient outcomes and program performance to facilitate continuous quality improvement.^55^

The HEARTS program has been successfully implemented in various regions, including the Americas, where it has been adapted by Pan American Health Organization (PAHO) as “HEARTS in the Americas” with the aim of tackling the increasing impact of cardiovascular disease.^58^ This regional adaptation focuses on improving hypertension control and CVD risk management through primary care-oriented management systems and clinical pathways. The program has demonstrated feasibility and acceptability, with significant improvements in hypertension control rates in participating countries.^55^

Key factors for the successful implementation of the HEARTS program include strong political support, integration into national health policies, and collaboration with international partners such as the Pan American Health Organization (PAHO) and other global health organizations. These partnerships provide technical expertise, funding, and capacity-building support to facilitate the program's expansion and sustainability.^29^

These drivers guide healthcare leaders and providers through a structured quality improvement process to achieve 80% diagnosis, 80% treatment, and 80% control among those with hypertension.^72^

A central element of HEARTS is team-based care, fostering collaboration among physicians, nurses, pharmacists, and other healthcare professionals to provide a holistic, patient-centered approach to managing hypertension and cardiovascular disease (CVD) risk.^73^^,^^74^ In this diverse American region, stroke prevention through team-based care includes clear roles, workflows, and accountability. Physicians focus on diagnosis, risk stratification, and treatment planning, while nurses, community health-workers, and other non-physician providers handle standardized blood pressure measurement, cardiovascular risk assessment, lifestyle counseling, medication titration under protocols, follow-up, and medication adherence support. This structured task-sharing method helps reduce fragmentation and prevent coordination failures that could otherwise weaken team-based care in prevention settings.

The initiative offers a practical, scalable clinical pathway aligned with the 2021 WHO hypertension guidelines.^61^^,^^70^ This pathway includes a standardized treatment protocol that recommends initiating treatment for individuals with blood pressure ≥140/90 mmHg, using a combination of two complementary classes of antihypertensive drugs. For individuals at high CVD risk, treatment is also recommended for those with systolic blood pressure ≥130 mmHg, along with the use of high-intensity statins to further reduce their CVD risk.^75^

To ensure accurate blood pressure measurement, HEARTS promotes the use of clinically validated automated measuring devices, alongside training and certification for healthcare providers to ensure consistent and reliable measurements across Primary Healthcare settings.^58^

Since its inception in 2016, the initiative has expanded significantly, currently involving 33 countries, with 12 countries scaling the model across their entire primary healthcare network. The standardized, high-quality clinical pathway has been approved in 28 countries, and the program currently covers 7158 primary healthcare centers, serving around 45 million adults, with 5 million receiving treatment. Notably, 65% of those treated have achieved hypertension control. By controlling hypertension—the primary risk factor for stroke—HEARTS offers a significant opportunity to reduce the burden of stroke and CVD across the region.

Key facilitators of successful implementation include strong political commitment from health ministries, endorsement by scientific societies, PAHO’s regional leadership, and alignment with primary health care strengthening and universal health coverage goals (https://www.paho.org/en/hearts-americas). Barriers encountered involve health system fragmentation, legal restrictions on professional scopes of practice, and limited health information systems for operational evaluation.^29^ Stroke Centers can be a powerful agent in implementing HEARTS and other preventive programs.

Building on the current HEARTS Clinical Pathway, HEARTS in the Americas is evolving into HEARTS 2.0—a major step forward in integrating comprehensive cardio-kidney-metabolic care at the primary health care level. A key milestone in this evolution is the identification of 45 evidence-based interventions designed to strengthen and update the HEARTS Clinical Pathway. Among the most promising is opportunistic screening for atrial fibrillation, particularly in individuals with high cardiovascular risk at any age, as well as all adults aged 65 and older. This marks a critical shift toward earlier detection and preventive strategies embedded in routine primary care.^76^

### Other examples of implementation programs in the Americas

Multiple programs have successfully lowered the risk of CVD and stroke in diverse health systems. Brazil’s Family Health Strategy is the backbone of the nation's primary health care system, delivering population-based care through multidisciplinary teams responsible for specific geographic areas. The team includes a physician, a nurse, nurse assistants, and community health-workers. Through task sharing, they deliver proactive prevention and chronic disease management with home-based follow-up, reducing stroke incidence and hospitalizations and improving control of major cardiovascular risk factors.^77^^,^^78^ Cities with ≥70% Family Health Strategy coverage have 18% lower stroke mortality (rate ratio 0.82, 95% CI 0.79–0.86) than cities without coverage. Complementing this national model, CARDIO4Cities in São Paulo showed multisectoral city action: a partnership between the Novartis Foundation and Municipal Health Secretariat. It integrated care pathways, community engagement, and performance monitoring. In districts adopting CARDIO4Cities, stroke hospitalizations dropped 54%, and stroke mortality decreased 43%.^79^ In parallel, the implementation of the HEARTS Program in Brazil began in Porto Alegre through a public–private partnership between Hospital Moinhos de Vento and the Municipal Health Secretariat. Using simplified protocols with free public medications, blood pressure measurement increased from 20% to 70%, improving detection of uncontrolled hypertension, new cases of hypertension, and previously undiagnosed atrial fibrillation.

In Chile, long-standing cardiovascular prevention programs—aligned with European and North American models but adapted locally—have successfully addressed a high burden of cardiovascular risk factors.^80^ These programs position Chile as a benchmark within the COTRACO study (Community-based model for management and follow-up by non-physician healthcare workers to improve awareness, treatment, and control of hypertension),^81^ where it serves as a usual-care comparison country while community-based, task-sharing hypertension interventions are implemented in Colombia and the Dominican Republic. This quasi-experimental study evaluates innovative workforce models. In Argentina, a feasibility study in low-income urban settings demonstrated that task shifting within primary care teams, combined with community health worker–led home interventions, can improve blood pressure control and is feasible and effective.^82^ Finally, Mexico’s “A Todo Corazón” program,^83^ led by the Mexican Social Security Institute, illustrates a comprehensive national approach that integrates primary and secondary prevention (addressing the high prevalence of obesity, hypertension, and diabetes), acute myocardial infarction management, and rehabilitation.

Other structured implementation programs in the Americas are listed in Supplementary Table S1. More detailed case examples are reported in Supplementary Table S2.

## Challenges and future directions

Tools to implement stroke prevention in the Americas are well-known and recognized in scientific literature. However, key obstacles hamper the implementation of these measures, primarily due to the limited capacity of health systems to address NCDs. Contributing factors include persistent health inequities, difficulties in managing modifiable risk factors, and barriers to effective community and population-based interventions.

Many regions, particularly low-income and rural areas, face a shortage of healthcare professionals and facilities equipped to provide comprehensive stroke prevention services.^84^ This is compounded by socioeconomic factors, such as poverty and low educational attainment, which limit public awareness and understanding of stroke risk factors and prevention strategies. Moreover, community-based interventions often face challenges in engagement, resource allocation, and achieving significant improvements in risk factor control. Addressing these challenges requires a coordinated effort to improve healthcare infrastructure, increase funding for public health initiatives and training of health professionals and community health-workers, and promote cross-border collaboration to share best practices and resources.

In looking towards the future of stroke prevention in the Americas, several promising directions emerge. A top priority should be enhancing regional collaboration to develop and implement evidence-based clinical practice guidelines, standardized treatment protocols, and comprehensive clinical pathways that will allow early diagnosis and appropriate treatment of stroke risk factors. These tools must be adaptable to local contexts to ensure consistency in prevention strategies across diverse healthcare systems, with a particular emphasis on primary care settings. Leveraging technology, such as telemedicine and mobile health applications, can significantly improve access to preventive care and education, particularly in remote and underserved areas.

Additionally, investing in community-based programs that emphasize lifestyle modifications, such as healthy eating and regular physical activity, can empower individuals to take proactive steps in reducing their stroke risk. Strengthening partnerships between governments, non-governmental organizations, and the private sector can facilitate the pooling of resources and expertise, driving innovation in prevention strategies. Furthermore, prioritizing research to better understand the unique epidemiological patterns of stroke in different populations will enable the development of targeted interventions.

A national stroke policy, integrated into Universal Health Coverage packages, can enable the widespread, coordinated implementation of a prevention program throughout the country. Moreover, stakeholders implementing public health strategies for stroke prevention are encouraged to report their results to prioritize interventions with the most impactful results in each region.

## Gaps in research

First, there is a limited understanding of the region-specific risk factors and epidemiological patterns of stroke, which vary widely due to diverse genetic, environmental, and lifestyle influences. As such, there is a need for comprehensive, large-scale studies that explore these variations to inform tailored prevention strategies. Moreover, evidence on the comparative effectiveness of various prevention strategies across different cultural and socioeconomic contexts is lacking. Second, there has been a suboptimal exploration of the role of social determinants of health, such as education, income, and access to healthcare, in stroke incidence and outcomes. Third, there is a need for more robust data on the long-term impact of emerging technologies, like telemedicine and digital health tools, for improving stroke prevention. Finally, ensuring the enrollment of diverse participants (in terms of sex, race, ethnicity, socioeconomic status, and geography) in pivotal clinical trials and studies should be a priority to ensure the applicability of results to marginalized and underrepresented populations.^19^ By addressing these key research gaps, stakeholders can develop more effective, equitable, and sustainable stroke prevention strategies across the Americas.

## Conclusion

Numerous opportunities exist to reduce stroke burden in the Americas. Perhaps the most important of all is strengthening health systems—particularly by improving access to and the quality of primary health care. This includes ensuring access to preventive interventions, effective medications, and high-quality services, all of which are essential for timely and equitable stroke prevention. Several initiatives have been proposed and are increasingly implemented in countries, with an emerging effort from global strategies to reduce the impact of risk factors in cardiovascular and cerebrovascular diseases. Health inequities, resource constraints, and a paucity of informed regional leaders constitute obstacles to the ideal implementation of stroke prevention in the region, especially in low- and middle-income countries. Large-scale epidemiological studies could provide a more comprehensive and ongoing actionable assessment of risk factor prevalence and control in the Americas.

## Contributors

SOM and PL conceived the idea and designed the paper. SOM analyzed the data, prepared the tables and figures, wrote the first draft, and conducted the final edit. PO contributed by suggesting literature, writing the paper, and conducting the final review. WVB reviewed the literature and contributed to the first draft. BO, VCU review and writing. All authors reviewed and provided input on manuscript drafts and approved the final version for publication. All authors have full access to all the data in the study, accept accountability for the overall work, and the corresponding author had the final responsibility for submitting the manuscript for publication.

## Data sharing statement

Upon reasonable request to the corresponding author, study data may be made available to other researchers.

## Declaration of interests

SOM: received research grants from the Brazilian Ministry of Health; received honoraria as speaker from Medtronic, Boehringer Ingelheim, Astra Zeneca, Bayer, Pfizer, Novartis, Novo Nordisk, Daiichi Sankyo, Servier, and Penumbra. VCU: received research grant from Genentech, INC. CA: received honoraria as Speaker from Boehringer Ingelheim; TFA: received honoraria as Speaker from Boehringer Ingelheim; PA: received honoraria as Speaker from Boehringer Ingelheim, Abbott, Ipsen, Boston Scientific and Knigth therapeutics; CSA: Receives Grants and fellowship from the National Health and Medical Research Council (NHMRC) of Australia, Medical Research Foundation of the UK, Consulting fees as Advisory Board for AstraZeneca Australia, is the Vice-President of the World Stroke Organization and the Editor-in-Chief of Cerebrovascular Diseases journal; PML: Received Research grant from Boehringer-Ingelheim, payment as part of Steering Committee from Johnson & Johnson and Advisory Board from Bristol Meyer Squib and Pfizer, honoraria for lectures from Pfizer, Angels educational events from Boehringer Ingelheim, Support for attending meetings and/or travel from the Iberoamerican Stroke Society and Global Stroke Initiative, President of the Chilean Stroke Association (ACEVE) and Vice-president of the Iberoamerican stroke society (SIECV); BO: Editor-in-Chief, Journal of the American Heart Association, President, Society for Equity Neuroscience, Member, World Stroke Organization Board; OPN: Received consulting fees from Boehringer-Ingelheim, Bristol-Myers-Squibb and Bayer, and speaker fees from Boehringer-Ingelheim, Editor-in-Chief, World Stroke Academy; GSS: Received grant from the Brazilian Ministry of Health, Consulting fees from Astrazeneca and Bayer, Payment or honoraria for lectures from Astrazeneca, Bard, Support for attending meetings from Boehringer Ingelheim; SA: Received payment or honoraria from Astra Zeneca and Silanes, Support for attending meetings from Astra Zeneca and Raffo, Participation on a Data Safety Monitoring Board from Astra Zeneca, LAC: Received grant from World Stroke Organization, Consulting fees from Allm Inc, IschemaView, AstraZeneca, Payment or honoraria for lectures from AstraZeneca, Boehringer Ingelheim, IschemaView, Support for attending Meetings from Boehringer Ingelheim, IschemaView, PMV: Received Research grants from ANID Fondecyt Regular 1221837 and Pfizer Research grant 76883481, Grant from Boehringer Ingelheim; AR: Participation on a Data Safety Monitoring Board or Advisory Board from Boston Scientific, Astra Zeneca, Shionogi, Brainomix, Chiesi. MJA received honoraria from Boehringer Ingelheim (Angels Initiative, Steering Committee); BA participated as consultant for NeuroDevice. JC received financial support, was speaker, participated in committee and was consultant for Boehringer Ingelheim. FC received financial support from Boehringer Ingelheim. RG was speaker for Novartis and IPSEN, and received financial support from Boehringer Ingelheim. CJ received honoraria from Boehringer Ingelheim and participated in committee for Boehringer Ingelheim (Angels Initiative Steering Committee) and WSO (Future Stroke Leaders). VN received honoraria from Boehringer Ingelheim. VVO received grant and financial support from Boehringer Ingelheim, and received honoraria from Novo Nordisk. JR received honoraria from Boehringer Ingelheim and Novo Nordisk. GS received a research grant from Roche. All other authors declare no conflicts of interest with the content of this manuscript.
