# Supplementary material for: Implementation of stroke prevention: a review of challenges and opportunities in the Americas
Source: Lancet Reg Health Am. 2026 May 22;58:101468. doi: 10.1016/j.lana.2026.101468 (PMC13280441; doi:10.1016/j.lana.2026.101468)
Supplement: Supplementary Tables S1 and S2 [file mmc1.docx]

**SUPPLEMENTARY MATERIAL**

**Running title: Implementation of stroke prevention: Challenges and opportunities in the Americas**

**TABLE OF CONTENTS**

1. Sup. Table 1. Structured Primary Prevention Programs in Americas………………………… 2

2. Sup. Table 2. Case studies and examples of implementation of prevention strategies in Latin American countries………………………………………………………………………………4

3. References…………………………………………………………………………………..…… 9

**Sup. Table 1. Structured Primary Prevention Programs in Americas**

| The initiative CARDIO4Cities | Among the limited initiatives targeting Latin America, CARDIO4Cities has raised important evidence towards population-based interventions. The CARDIO4Cities is a multisectorial urban cardiovascular health strategy aimed at improving primary prevention of CVDs focused on LMIC. This initiative has been successfully implemented in cities such as Dakar, Senegal and Sao Paulo, with a focus on mitigating the burden of stroke through optimized hypertension treatment and risk reduction strategies. CARDIO4Cities included not only medical treatment of hypertension, but also community-based advocacy and engagement, ultimately leading to healthcare strengthening.  The CARDIO4Cities results provided compelling evidence towards a structured approach to reduce cardiovascular risk factors in large cities. Specifically, in Sao Paulo, the districts where the strategy was implemented experienced a 54% reduction in stroke hospitalization rates, and 43% reduction of death from stroke(1).  In São Paulo, the initiative was implemented through a strategic partnership between the Novartis Foundation and the Municipal Health Secretariat, combining technical support and global expertise with public-sector leadership. The Novartis Foundation supported evidence-based hypertension strategies, while the Health Secretariat led territorial implementation and integration within primary health care, enabling population-level impact on cardiovascular and stroke prevention. In the districts adopting Cardio4Cities experienced a 54% reduction in stroke hospitalisations and a 43% reduction in stroke mortality, alongside improved blood pressure (BP) control, demonstrating the potential of coordinated, intersectoral approaches to reduce cardiovascular and stroke burden at scale(2). |
| --- | --- |
| American Heart Association prevention program | Focused on systemic changes in healthcare delivery in the various healthcare settings, the American Heart Association (AHA) prevention program(3) comprehends the integration of different levels of care, from individual-level (multidisciplinary collaboration, healthcare technology), community-level (community engagement) and government-level (health-related policies) approaches. By leveraging team-based care and community health workers, the program enhances patient engagement and adherence to treatment, further supporting CVD prevention efforts in the region. |
| The Million Hearts® 2027 (Center for Disease Control) | This initiative aims to prevent one million cardiovascular events by 2027 through coordinated efforts in clinical and community settings, focusing on evidence-based strategies such as hypertension control, tobacco cessation, and cholesterol management(4). While the program is U.S.-based, its comprehensive, population-level approach offers valuable insights for Latin American countries, where cardiovascular disease remains a leading cause of morbidity and mortality. Adapting elements of Million Hearts, such as standardized treatment protocols and public health campaigns, could enhance the effectiveness of prevention strategies in Latin America, especially in resource-limited settings. |

**Sup. Table 2. Case studies and examples of implementation of prevention strategies in Latin American countries.**

| **Country** | **Examples** |
| --- | --- |
| Brazil | Several cardiovascular prevention strategies have been implemented in Brazil, focusing on different aspects of cardiovascular health. One notable example is the Brazilian Cardioprotective Nutritional Program (BALANCE), which aimed to improve dietary quality and provide secondary prevention of cardiovascular events through nutritional education tailored to regional foods(5). Despite its innovative approach, the program showed only slight improvements in diet adherence and did not significantly affect cardiovascular event rates or mortality, but holds great potential for long-term implementation in the public health system.  Another significant initiative is the Family Health Strategy (FHS), which is Brazil's largest primary healthcare program, delivering continuous, population-based care through multidisciplinary teams, including a physician, nurse, nurse assistant, and community health workers responsible for specific geographic areas. FHS teams provide proactive prevention, manage chronic diseases, promote health, and conduct home follow-ups, all while engaging the community effectively and ensuring continuity of care. It has been associated with significant reductions in cardiovascular mortality, stroke incidence, and avoidable hospitalizations, as well as improved control of hypertension, diabetes, and other vascular risk factors(6,7) . By combining task sharing, community outreach, and systematic risk-factor management, the FHS represents a scalable and effective model for equitable cardiovascular and stroke prevention in middle-income settings.  Additionally, many other non-government initiatives have arisen lately in Brazil. As mentioned above, the successful CARDIO4Cities initiative in São Paulo represents a multisectoral approach to hypertension care, aiming to improve blood pressure control through a series of interventions developed with local and international stakeholders. This initiative has shown improvements in blood pressure control among treated patients, highlighting the potential of intersectoral collaboration in managing cardiovascular risk(2).  The implementation of the HEARTS Program in Porto Alegre, a city in the southern part of Brazil, was a public-private partnership between Hospital Moinhos de Vento and the Health Secretary of Porto Alegre. All 133 Primary Care Units were trained in the pathways and the simplified protocols, which were adapted to utilize free medication available in Brazil. Additionally, risk stratification was implemented through the HEARTS Calculator. Before the program, only 20% of patients treated in the unit had their blood pressure measured. After the program's implementation, 70% of all individuals treated had their BP measured. With software for triage and patient care guidance through the protocols, 34% of hypertensive patients with BP > 140/90 mmHg were detected, along with 11% of new hypertensive patients and 2% of patients with atrial fibrillation who had not received a previous diagnosis. The program is now expanding to the entire state. |
| Panama | In Panama, the implementation of cardiovascular prevention strategies has been influenced by regional initiatives such as the HEARTS in the Americas program(8,9). Although specific details about Panama's unique implementation were not published, the country's participation in such regional initiatives suggests a commitment to improving cardiovascular health through structured, evidence-based approaches. |
| Chile | In Chile, cardiovascular prevention strategies have been tailored to address the high prevalence of cardiovascular risk factors, similar to those seen in European and North American populations. Successful primary and secondary prevention programs have been developed to manage these risk factors, including hypertension and hypercholesterolemia, through both pharmacological and lifestyle interventions(10) [(9)](https://paperpile.com/c/gIeBq3/D896). These programs are designed to reduce the incidence of cardiovascular events and mortality by applying criteria similar to those used in North America and Europe, with slight modifications to suit the local context. Additionally, the COTRACO study (Community-based model for management and follow-up by non-physician healthcare workers to improve awareness, treatment, and control of hypertension) is a quasi-experimental, community-based intervention inspired in HOPE4 study, evaluating hypertension management delivered by non-physician health workers through standardized treatment protocols, lifestyle counselling, and medication to enhance BP control in low- and middle-income settings. While implementing the intervention in Colombia and the Dominican Republic, Chile serves as a usual-care comparison country. This reflects its existing cardiovascular prevention programs and offers a benchmark for evaluating new task-sharing models designed to lower hypertension-related cardiovascular and stroke risks(11). |
| Argentina | In Argentina, a feasibility study evaluated a multi-component intervention to improve the detection and treatment of CVD risk factors in poor urban settings. This intervention involved task shifting among primary care clinic staff, training for healthcare providers, and home-based interventions delivered by community health workers. The study showed improvements in clinical outcomes, such as blood pressure control, and demonstrated the feasibility and acceptability of the intervention in low-income communities(12). |
| Colombia | In Colombia, the "De Todo Corazón" (DTC) program is a notable cardiovascular prevention strategy. This program targets low-income populations with conditions such as hypertension, diabetes mellitus, chronic kidney disease, and dyslipidemia. It emphasizes adherence to medical appointments and control of cardiovascular risk factors, which has been shown to significantly reduce the risk of cardiovascular outcomes like stroke and myocardial infarction in adherent patients(13). Additionally, the COTRACO study involving Chile is also recruiting participants in Colombia to explore a community-based model using non-physician healthcare workers to improve hypertension management, which could enhance awareness, treatment, and control of hypertension(11). |
| Mexico | "A Todo Corazón" program in Mexico is a comprehensive care program developed by the Mexican Social Security Institute (IMSS). This program addresses the prevention, treatment, and rehabilitation of cardiovascular diseases through a multi-faceted approach. It includes promotion and primary prevention of CVDs, a national strategy for managing acute myocardial infarction, and secondary prevention and rehabilitation efforts. The program aims to strengthen actions to mitigate the impact of CVDs in Mexico, which has a high prevalence of risk factors such as obesity, hypertension, and diabetes(14). |
| Peru | In Peru, while specific cardiovascular prevention programs are not detailed in the provided literature, the country participates in regional initiatives like the HEARTS in the Americas program(8, 9), which aims to improve hypertension control and cardiovascular risk management in primary care settings. This program focuses on implementing standardized treatment protocols and enhancing healthcare delivery systems to manage cardiovascular risk factors effectively. |
| Uruguay | In Uruguay, cardiovascular prevention efforts are often integrated into broader public health strategies. The country has been involved in initiatives to improve the management of cardiovascular diseases through primary healthcare reforms and the implementation of evidence-based guidelines for hypertension and other cardiovascular risk factors. These efforts are part of a larger regional focus on optimizing cardiovascular disease prevention and management across the Americas(15). |
| Guatemala | The Guatemalan Ministry of Health has recently conducted a pilot trial to assess the feasibility and acceptability of the WHO HEARTS program(16). Two districts were selected to evaluate the package of five HEARTS-aligned implementation strategies to improve the pharmacological treatment of hypertension and diabetes. Results were promising and indicated that the program was feasible and accepted, though some adjustments should be made to increase long-term adherence. Next steps may include a plan to scale up the implementation of preventive strategies to national coverage. |

**REFERENCES**

1.Ferrer JME, Boch J, Aerts A, Anne M, Avezum A, Barboza J, et al. Stroke Outcomes in a Population-Focused Urban Hypertension Program in Brazil and Senegal. J Am Heart Assoc. 2025 May 6;14(9):e038816.

2. Avezum Á, Drager LF, Reiker T, Bigoni A, Leonel LP, Abreu A, et al. An Intersectoral Approach to Hypertension Care: Solutions for Improving Blood Pressure Control in São Paulo, Brazil. Am J Hypertens. 2024 Apr 15;37(5):366–78.

3. Agarwala A, Patel J, Stephens J, Roberson S, Scott J, Beckie T, et al. Implementation of Prevention Science to Eliminate Health Care Inequities in Achieving Cardiovascular Health: A Scientific Statement From the American Heart Association. Circulation. 2023 Oct 10;148(15):1183–93.

4. Million Hearts 2027 [Internet]. [cited 2025 May 25]. Million Hearts 2027. Available from: <https://millionhearts.hhs.gov/about-million-hearts/index.html>

5. Weber B, Bersch-Ferreira ÂC, Torreglosa CR, Marcadenti A, Lara ES, da Silva JT, et al. Implementation of a Brazilian Cardioprotective Nutritional (BALANCE) Program for improvement on quality of diet and secondary prevention of cardiovascular events: A randomized, multicenter trial. Am Heart J. 2019 Sep;215:187–97.

6. Ortega F, Pele A. Brazil’s unified health system: 35 years and future challenges. Lancet Reg Health Am. 2023 Dec;28:100631.

7. Rasella D, Harhay MO, Pamponet ML, Aquino R, Barreto ML. Impact of primary health care on mortality from heart and cerebrovascular diseases in Brazil: a nationwide analysis of longitudinal data. BMJ. 2014 Jul 3;349:g4014.

8. Ordunez P, Campbell NRC, DiPette DJ, Jaffe MG, Rosende A, Martinez R, Gamarra A, Lombardi C, Parra N, Rodriguez L, Rodriguez Y, Brettler J. HEARTS in the Americas: Targeting Health System Change to Improve Population Hypertension Control. Curr Hypertens Rep. 2024 Apr;26(4):141-156. doi: 10.1007/s11906-023-01286-w. Epub 2023 Dec 2. PMID: 38041725; PMCID: PMC10904446.

9. Moran AE, Gupta R; Global Hearts Initiative Collaborators. Implementation of Global Hearts Hypertension Control Programs in 32 Low- and Middle-Income Countries: JACC International. J Am Coll Cardiol. 2023 Nov 7;82(19):1868-1884. doi: 10.1016/j.jacc.2023.08.043. Epub 2023 Sep 19. PMID: 37734459.

10. Bunout D, Escobar E. [Prevention of cardiovascular diseases: should the same criteria be applied in Latin America and Europe and North America?]. Rev Esp Cardiol. 2000 Jul;53(7):889–95.

11. Lora Mantilla AJ, Parra Gomez LA, Camacho-López PA, Otero-Wandurraga J, Novella B, González-Medina A, et al. Community-based model for management and follow-up by non-physician healthcare workers to improve awareness, treatment, and control of hypertension: The COTRACO study protocol. Heliyon. 2025 Jan 30;11(2):e41726.

12. Rosana P, Danaei G, Gutierrez L, Cavallo A, Lopez MV, Irazola V. An innovative approach to improve the detection and treatment of risk factors in poor urban settings: a feasibility study in Argentina. BMC Public Health. 2021 Mar 22;21(1):567.

13. Miranda-Machado P, Salcedo-Mejía F, Parra-Padilla D, Vargas-Moranth R, Alvis-Zakzuk NR, Paz-Wilches J, et al. Effectiveness of a prevention program in the incidence of cardiovascular outcomes in a low-income population from Colombia: a real-world propensity score-matched cohort study. BMC Public Health. 2020 Sep 17;20(1):1416.

14. Borrayo Sanchez G, Rosas Peralta M, Martínez Montañez OG, Justiniano Cordero S, Fajardo Dolci G, Sepulveda Vildosola AC, et al. Implementation of a Nationwide Strategy for the Prevention, Treatment, and Rehabilitation of Cardiovascular Disease “A Todo Corazón.” Arch Med Res. 2018 Nov;49(8):598–608.

15. Schwalm JD, Joseph P, Leong D, Lopez-Lopez JP, Onuma O, Bhatt P, et al. Cardiovascular disease in the Americas: optimizing primary and secondary prevention of cardiovascular disease series: cardiovascular disease in the Americas. Lancet Reg Health Am. 2025 Feb;42:100964.

16. Wellmann IA, Ayala LF, Valley TM, Irazola V, Huffman MD, Heisler M, et al. Evaluating the World Health Organization’s Hearts Model for Hypertension and Diabetes Management: A Pilot Implementation Study in Guatemala. Glob Heart. 2025 Jan 31;20(1):9.
